# Hygiene of Old Age

**Published:** 1893-02

**Authors:** 


// HALL'S
### JOURNAL OF HEALTH

TRUTH DEMANDS NO SACRIFICE; ERROR CAN MAKE NONE.

Vol. 40. FEBRUARY, 1893. No. 2.

### HYGIENE OF OLD AGE.

A person of advanced years no longer needs food to promote the growth of tissue, for tissue growth in him has long since ceased. In his present condition of diminished activity, the waste of his tissues is also greatly lessened, and the need of food to repair this waste is correspondingly less. To maintain the vital heat is still his urgent need, and with increasing years the task grows more and more difficult.

But as respects senility, no two persons are alike. Some approach it at fifty, while others scarcely feel it at eighty. In a word those who have lived fast and irregular lives, are older in all the ills that prey upon age, than others of correct habits at two or three additional decades.

Not only do the old require less food than the young or middle aged, but food of a somewhat different character. Flesh food, and especially lean meat, which is chiefly useful in promoting the growth and repairing the waste of tissue, should not form a large part of the old man's diet. And observe how perfectly nature has adapted his capabilities to his needs. The teeth, which are required most of all in tearing and grinding the fibre of meat, to fit it for digestion, have now disappeared, or become so weak and decayed as to be unfit for performing this office. Errors in diet are specially harmful now. The young man has a reserve fund of vitality to draw upon, and though he may suffer acutely for a time, when too much food is taken, or food of an improper character or not properly prepared, yet he soon throws it off and does not seem to suffer permanently. But the old man's capacity in this regard has diminished with the increase of years.

The extravagances of over-eating which in early life might have only caused him a day's discomfort from indigestion, or of misery from a bilious attack, would now be liable to result in sudden death. He must therefore carefully measure his digestive power, and adapt his food, both in quantity and kind, to his actual needs.

To enable him to reach this end in safety ; to adapt his habits meanwhile to his changed conditions ; to conserve his strength and favor his weaknesses, and thus to conduct him safely through the dangers incident to advancing years, and bring him to the close of life by as easy a road as possible, with the greatest amount of comfort and the least of suffering, are the objects of the hygiene of old age, and since large quantities of food burden the stomach and oppress the system, it is better, in old age as in infancy—in man's second childhood as in his first—that he should take food not only in much smaller quantities than in middle life, but also at more frequent intervals.

No fact is better established than the need of abundant exercise, both physical and mental, to the prolongation of life, health and vigor. Few things are more disastrous to these ends, than for a man in advanced years, accustomed to a stirring and active life, abruptly to "retire from business," thereby exchanging habits of labor for those of ease, of a freedom from care, under the mistaken notion of thereby enjoying a well-earned rest for the remainder of his days. Rather should his relinquishment of business be gradual, with his lessening duties adapted to the failing energies of body and mind, but always sufficient to preserve his interest in life, and incite him to a reasonable degree of exertion. Not only is it "better to wear out than to rust out," but it takes longer to do it. If his business is such as to keep him much of the time out of doors, so much the better for his health. If not, then he needs some additional incentive to lead him into the pure air and sunshine, essential to both age and youth.

The power in age of resisting external influence, which in youth was strong, is now almost gone. He needs, therefore, to use special care to protect himself from heat, cold and atmospheric vicissitudes, for these are responsible for a very large proportion of what may be called premature or accidental deaths among the aged. Especially, is cold a mortal foe to old age.

Add to the effects of cold those of heat, moisture, winds and sudden changes of temperature, and we have, in a climate like ours, a most formidable array of dangers to old age from atmospheric causes. To guard against these, the old man must not only suit his food to the climate and season, but he must clothe himself warmly—preferably in woolen garments, as being the poorest conductor of heat—must avoid all undue exposure either to extreme or sudden changes of temperature, and must occupy a comfortable room. His sleeping rooms should be warm, well-aired and dry. Many a time has the "spare room" proven fatal to gray hairs and decripit old age.

Statistics show that more women than men have become old. It may reasonably be supposed that a part of the superior longevity of women is due to her more quiet, regular and temperate life, less injury from passion and excitement, and less exposure to atmospheric vicissitudes. If this be the case, it furnishes a valuable insight as to the kind of life which should be followed, not only by those who would grow old, but by those who, having already reached advanced life, desire still further to prolong their days.

The integrity of the heart and nervous system demands that the old man should avoid all extreme or sudden physical exertion, all intense and depressing mental emotion. Running to catch cars, lifting a heavy weight, making an eloquent and impassioned after-dinner speech, or indulging in a paroxysm of passion—all of these have often been proven to be only forms of suicide for the weakened heart and brittle arteries of the aged. The safety of gray hairs depends rather upon the regular continuance in accustomed paths, where to go on is easier than to stop or turn aside. Habits are strong in the decline of life, and not easily changed. To act in accordance with these is to travel in the line of the least resistance.

The man who has ceased to take an active concern in what is going on in the world about him, has but a feeble hold upon the world itself. When the wish and the will to live are gone, life is sure soon to follow.

A cheerful disposition, which enables its possessor to see the bright side of everything, and prevents him from wearing himself out with worry when things do not go to suit him, is a potent factor in prolonging life. Mental activity, if not coupled with too much nervous strain, may with advantage be kept up to the close of life. It is a well known fact that literary and scientific men, are as a rule long lived. In all countries, ministers among professional men, and farmers among manual laborers, are the longest lived classes in the commuuity, and they are exactly the ones who enjoy the benefits of mental and physical labor under the most favorable conditions.

Especially does old age need abundant sleep, that all the vital forces may be carefully husbanded.

